# Inhibition of keloid by ^32^P isotope radiotherapy through suppressing TGF-β/Smad signaling pathway

**DOI:** 10.18632/aging.205160

**Published:** 2023-10-31

**Authors:** Long Xie, Liqun Huang, Guanjie Zhang, Yingrui Su

**Affiliations:** 1 Department of Nuclear Medicine, The Second Affiliated Hospital of Fujian Medical University, Quanzhou, China

**Keywords:** keloid, TGF-β/Smad, ^32^P isotope, Coll1α1, SRI-011381

## Abstract

Background: Keloid seriously affects the appearance, and is accompanied by some symptoms including pain, burning, itching. Radioactive nuclides such as ^32^P have been proved to be effective in inhibiting the formation of keloid, but the mechanism remains unclear.

Methods: The keloid animal model was established through keloid tissues implantation. Hematoxylin-Eosin (HE) and Masson staining were performed to investigate histological changes and collagen deposition. The mRNA and protein expression were assessed using RT-PCR and western blotting, respectively. Cell apoptosis and cycle were evaluated through flow cytometry.

Results: Both ^32^P isotope injection and skin path significantly reduced the size of keloid, and inhibited TGF-β/Smad signaling pathway. SRI-011381, the agonist of TGF-β/Smad signaling pathway, markedly reversed the influence of ^32^P isotope on cell proliferation, cell apoptosis, cell cycle of LNCaP cells and TGF-β/Smad signaling pathway.

Conclusions: ^32^P isotope injection and skin path greatly reduced the size of keloid, and the TGF-β/Smad signaling pathway was remarkably inhibited by ^32^P isotope treatment. The regulation of dermal fibroblast by ^32^P isotope was reversed by SRI-011381. ^32^P isotope might inhibit keloid through suppressing TGF-β/Smad signaling pathway. Our study provides a novel therapeutic strategy for the treatment of keloid.

## INTRODUCTION

Keloid is a benign skin disease, but it often seriously affects the appearance, and can be accompanied by pain, burning, itching and other symptoms, and even affect the function of the limbs, which has a great negative impact on life [1]. In the process of skin wound healing, keloid, due to the interaction of molecules, cells, physiology, biochemistry, physics and other factors, leads to the imbalance of body repair mechanism, abnormal keloid tissue grows excessively [2], gradually bulges from the skin surface, exceeds the original damage range, and invades the surrounding normal tissue without natural regression [3]. At present, the treatment methods are roughly divided into surgical treatment, laser therapy, physical therapy, and drug therapy [4]. Each of the above treatment methods has its own advantages and has a certain inhibitory effect on scars, but they all have shortcomings, some of which have little effect, some are expensive, and the clinical effect is not ideal [5, 6].

Using radioactive nuclides such as ^32^P to release β-radiation can significantly inhibit scar hyperplasia and is one of the best treatment methods [7]. Purely released β-rays can treat keloid by influencing the activity of fibroblasts, inhibiting the synthesis of collagen fibers, blocking microvessels, and reducing the blood supply to keloid tissue through electric radiation [8]. Among them, the use cost of ^32^P is the lowest, making it suitable for clinical treatment. The total treatment course is relatively cumbersome, and other relevant clinical departments still have little understanding and application [9]. Therefore, this study aims to study a more effective ^32^P treatment plan within a safe range, in order to further promote its clinical application and understand possible treatment mechanisms.

Keloid is a benign skin tumor beyond the original damage range formed by excessive hyperplasia and hyaline degeneration of skin connective tissue [10]. It has gene susceptibility, epigenetic regulation, and is of great significance to gene expression, regulation, and inheritance, including block non coding RNA, histone modification, etc. [11]. Activating or inhibiting TGF-β/Smad can induce or inhibit fibroblast proliferation and collagen synthesis, and promote or inhibit the formation of keloid [12].

The TGF-β/Smad signaling pathway plays a crucial role in keloid formation, contributing to the excessive and abnormal deposition of extracellular matrix (ECM) components, which characterizes this fibrotic skin disorder [13]. Keloids are an aberrant response to wound healing, where an overproduction of collagen and other ECM proteins leads to the formation of raised, thickened, and fibrous scars that extend beyond the original wound site [14]. Whether ^32^P isotope radiotherapy could regulate keloid has not been reported.

## RESULTS

### Both ^32^P isotope injection and skin path significantly reduced the size of keloid

**Figure 1:**
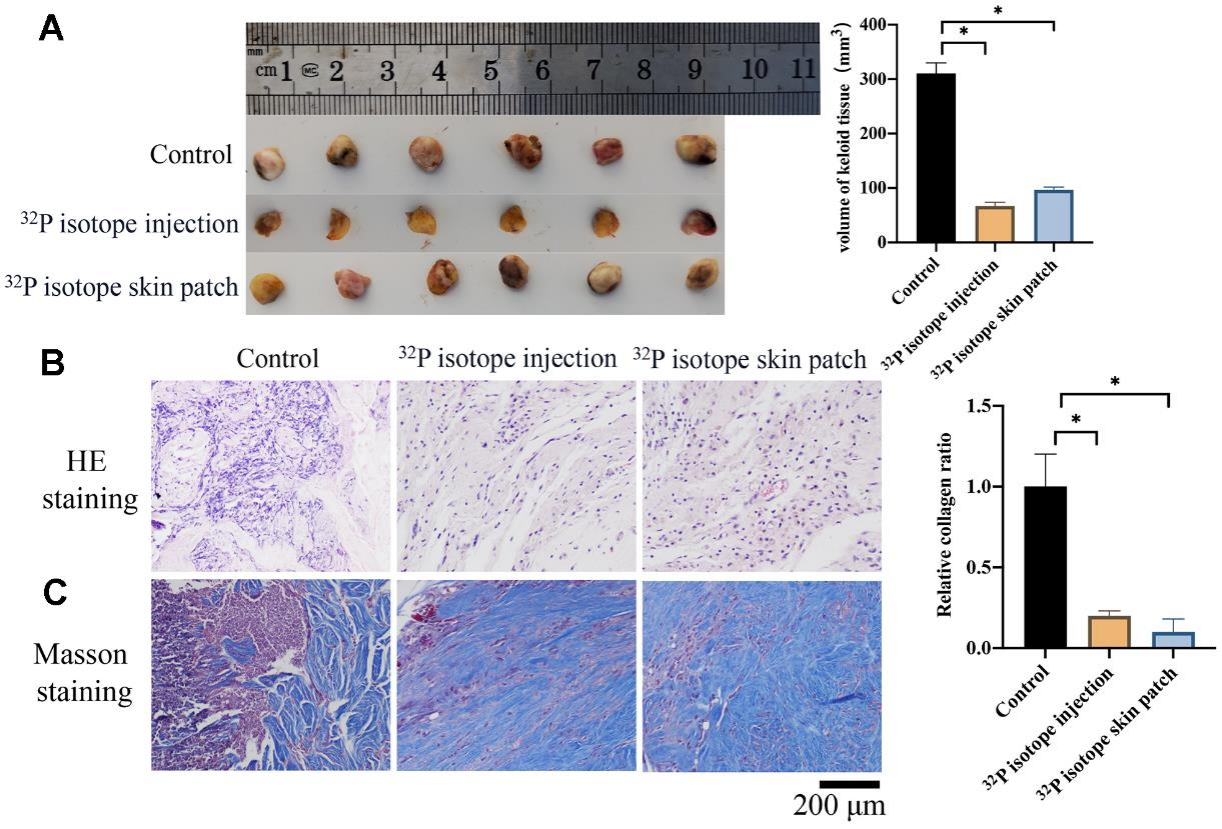
**Both ^32^P isotope injection and skin path significantly reduce the size of keloid.** (**A**) ^32^P isotope injection and skin path significantly reduced the size of keloid (n=6); (**B**) HE staining was performed to investigate the histological changes (n=3); (**C**) Masson's trichrome staining was performed to investigate the collagen deposition (n=3). * indicates p <0.05.

Two types of treatments, ^32^P isotope injection and ^32^P isotope skin path, were applied to treat keloid. Both ^32^P isotope injection and skin path significantly reduced the size of keloid (Figure 1A). HE staining was performed to investigate the histological changes. The disorder tissues were improved after ^32^P isotope injection and ^32^P isotope skin path treatment (Figure 1B). Masson's trichrome staining (Figure 1C) revealed that ^32^P isotope injection and ^32^P isotope skin path treatment remarkably suppressed the collagen deposition.

### Both ^32^P isotope injection and skin path significantly inhibited TGF-β/Smad signaling pathway

**Figure 2:**
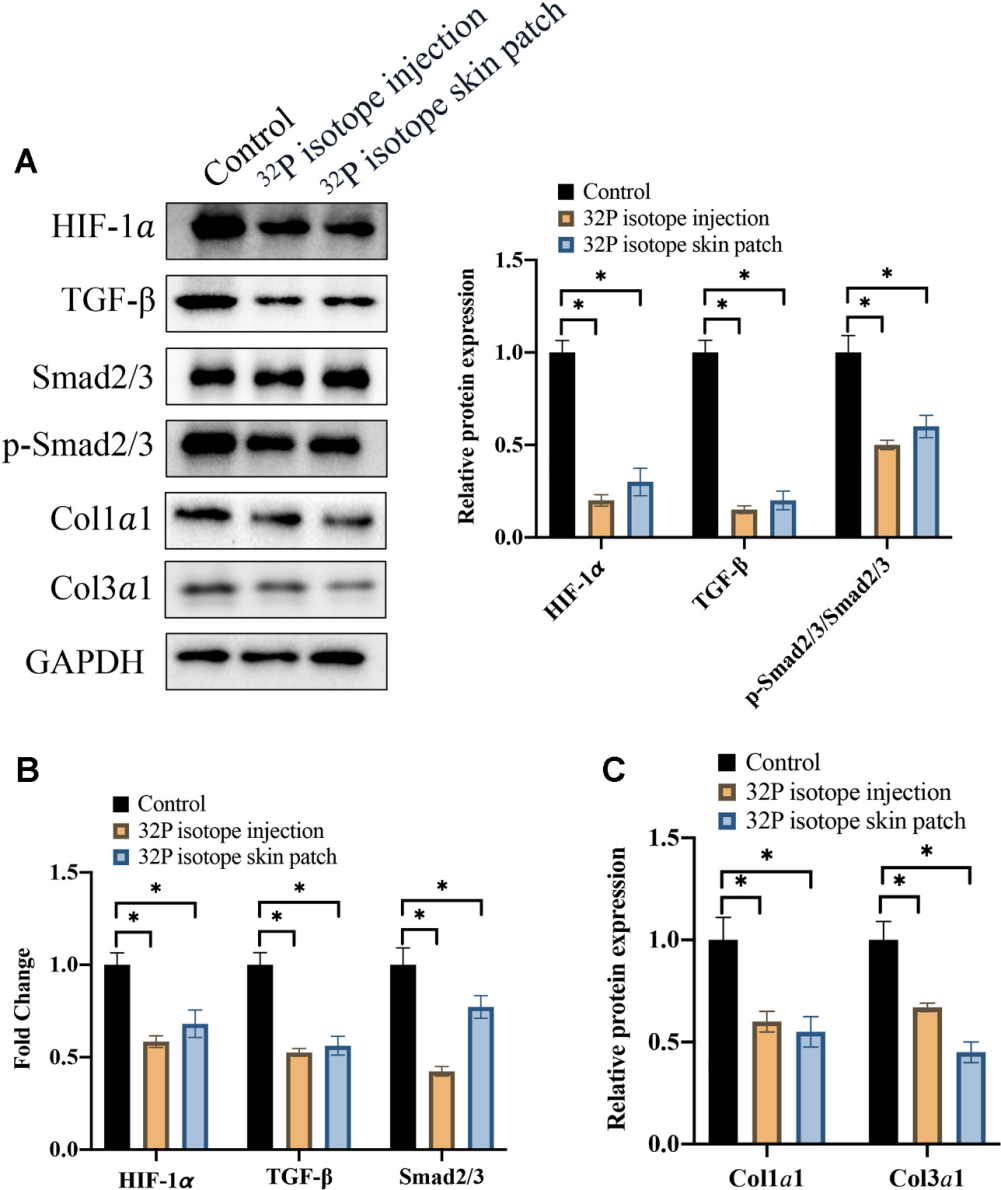
**Both ^32^P isotope injection and skin path significantly inhibited TGF-β/Smad signaling pathway.** (**A**) The protein expression of TGF-β/Smad signaling pathway was measured with western blotting (n=3); (**B**) The mRNA levels of HIF-1α and TGF-β/Smad signaling pathway were analyzed (n=3); (**C**) The protein levels of Coll1α1 and Coll3α1 were analyzed (n=3). * indicates p <0.05.

HIF-1α and TGF-β/Smad signaling pathway are closely linked with the formation of keloid. Coll1α1 and Coll3α1 are representative markers of collagen deposition. We found that Both ^32^P isotope injection and skin path treatments could markedly inhibit the protein and mRNA expression of HIF-1α, TGF-β, p-Smad2/3/Smad2/3 (Figure 2A, 2B), Coll1α1, and Coll3α1 (Figure 2A, 2C). Therefore, ^32^P isotope treatment might inhibit keloid through targeting TGF-β/Smad signaling pathway.

### Both ^32^P isotope injection and skin path significantly promoted cell apoptosis and regulated cell cycle

**Figure 3:**
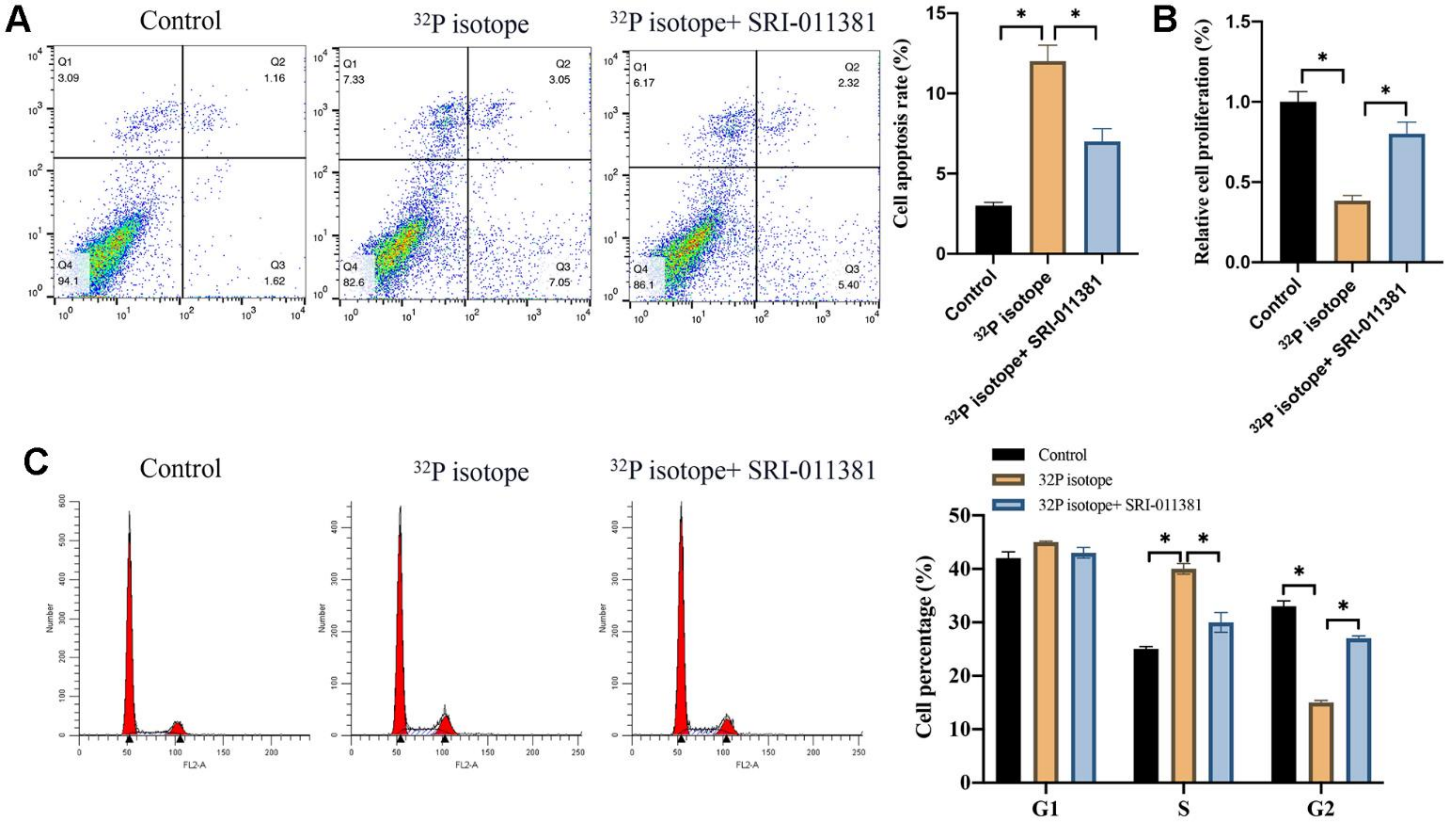
**Both ^32^P isotope injection and skin path significantly promoted cell apoptosis and regulated cell cycle.** (**A**) The cell apoptosis was measured with flow cytometry (n=3); (**B**) The cell proliferation ability was detected with CCK8 assay (n=3); (**C**) The cell cycle change was measured with flow cytometry (n=3). * indicates p <0.05.

To investigate if ^32^P isotope treatment could affect keloid through targeting TGF-β/Smad signaling pathway. The agonist of TGF-β/Smad signaling pathway, SRI-011381, was used. We found that the ^32^P isotope greatly increased cell apoptosis, inhibited cell proliferation, arrested cells in the S stage while reduced the cell percentage in the G2 stage (Figure 3A–3C). However, simultaneous treatment with SRI-011381 significantly reversed the effects of ^32^P isotope, leading to a decreased cell apoptosis, increased cell proliferation and cell percentage in the G2 stage (Figure 3A–3C). We demonstrated that activation of TGF-β/Smad signaling pathway *in vitro* could remarkably reverse the influence of ^32^P isotope.

### C16PAF markedly reversed the influence of ^32^P isotope on cell apoptosis of LNCaP cells and TGF-β/Smad signaling pathway

**Figure 4:**
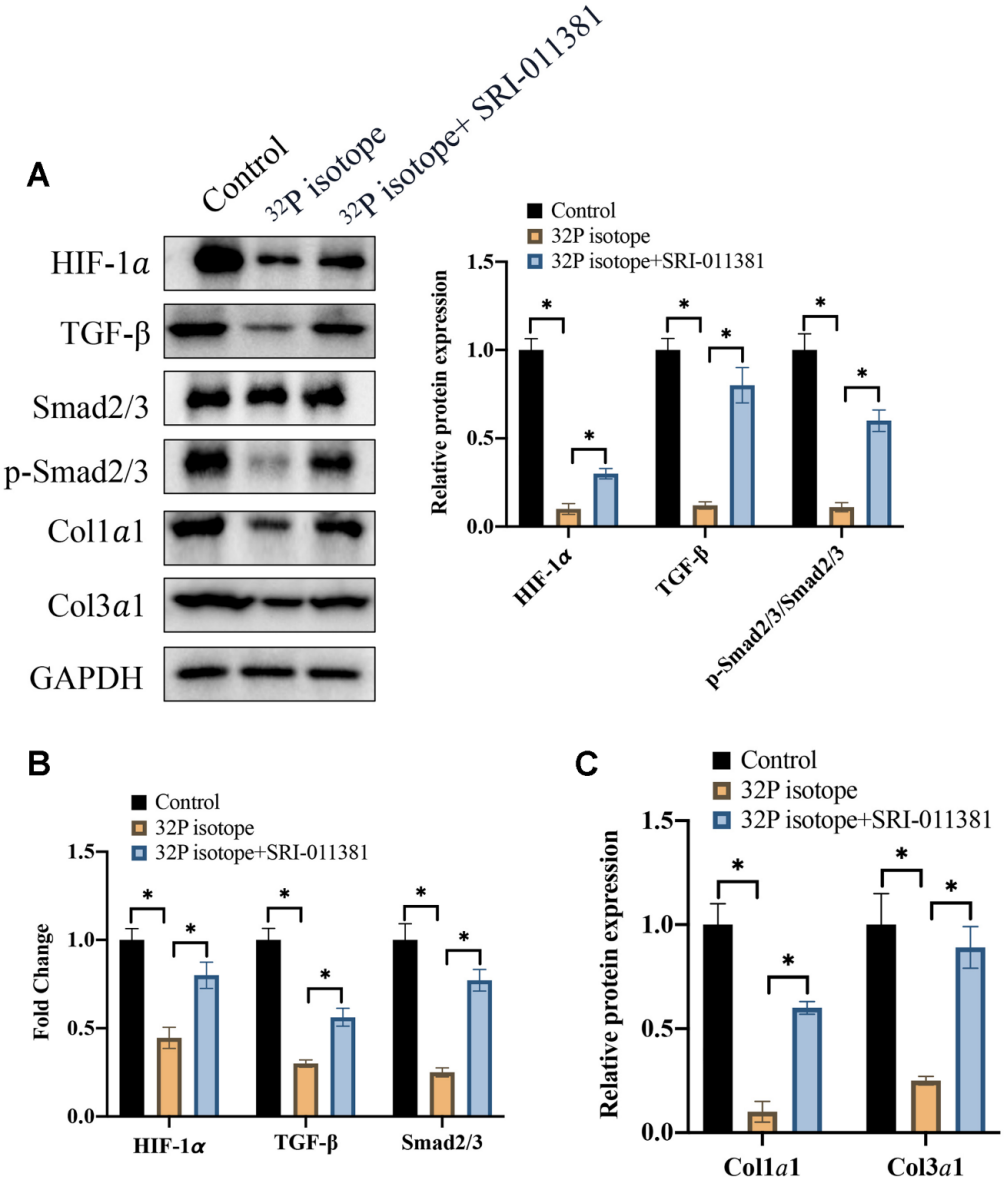
**C16PAF markedly reversed the influence of ^32^P isotope on cell apoptosis of LNCaP cells and EMT process.** (**A**) The protein expression of TGF-β/Smad signaling pathway was measured with western blotting (n=3); (**B**) The mRNA levels of HIF-1α and TGF-β/Smad signaling pathway were analyzed (n=3); (**C**) The protein levels of Coll1α1 and Coll3α1 were analyzed (n=3). * indicates p <0.05.

In addition, the influence of ^32^P isotope treatment and SRI-011381 on the protein and mRNA expression of HIF-1α, TGF-β/Smad2/3, Coll1α1, and Coll3α1 were also measured. We demonstrated that the inhibitions of HIF-1α, TGF-β, p-Smad2/3/Smad2/3 by ^32^P isotope were greatly reversed after SRI-011381 treatment (Figure 4A–4C).

## DISCUSSION

The prevalence of keloid in the population is about 4.5-16%, often accompanied by uncomfortable symptoms [15]. ^32^P radionuclide brachytherapy has a good aesthetic treatment effect on scars, with low recurrence rate, low complications, high patient satisfaction and comfort, which can greatly alleviate the physical and mental injuries caused by the disease [16]. It is an effective treatment method that can be widely used in clinical practice, and has a good market prospect.

In the process of skin healing, fibroblasts proliferate excessively and secrete a large amount of extracellular matrix, and cell apoptosis is delayed and degradation is insufficient, and local microvascular formation is increased, many factors promote its gradual formation of keloid [17, 18]. ^32^P released β-rays can directly or indirectly ionize and destroy DNA molecular structure, inhibit cell division and proliferation, inhibit fibroblast migration, proliferation and collagen synthesis and secretion, weaken immune cell function and reduce dysfunctional blood vessels, strongly inhibit inflammatory reaction, and can also effectively induce cell apoptosis and reduce TGF-β [19], Reduce the synthesis of extracellular matrix and collagen fiber, thus inhibiting the formation of keloid. Other kinds of radiotherapies treating keloid have been reported. X-ray, cobalt-60, and linear accelerator have been reported to treat keloid [20]. Meanwhile, brachytherapy may significantly reduce the recurrence of keloid compared with X-ray or electron beam [21]. The overall complication rate of radiotherapy for keloids was 19% including acute complications (erythema, wound dehiscence, infection, and peeling) and chronic complications (skin color changes, telangiectasia, and fibrosis) [22]. In this research, the complications described above were not observed.

During the normal wound healing process, TGF-β, a multifunctional cytokine, plays a pivotal role in promoting tissue repair and ECM synthesis. However, in keloid formation, the TGF-β signaling pathway becomes dysregulated, leading to an excessive and prolonged activation of downstream Smad proteins [23]. The dysregulation of the TGF-β/Smad signaling pathway in keloid formation leads to the abnormal and excessive deposition of ECM proteins, resulting in the development of fibrotic scars [24]. Understanding the molecular mechanisms underlying this process may pave the way for the development of targeted therapies aimed at modulating TGF-β/Smad signaling to mitigate keloid formation and improve patient outcomes [25]. In the present study, we found that Both ^32^P isotope injection and skin path significantly inhibited TGF-β/Smad signaling pathway. Meanwhile, the regulation of dermal fibroblast by ^32^P isotope was reversed by the agonist of TGF-β/Smad signaling pathway, SRI-011381. These findings indicate that ^32^P isotope might inhibit keloid through suppressing TGF-β/Smad signaling pathway.

However, our research lacks further investigation about the function mechanism of ^32^P isotope in inhibiting keloid. In recent years, studies have found that genes related to tumor growth in Keloid lesions may be abnormal. MicroRNAs (miRNAs) expression disorder may promote the development of keloid [26]. MiRNAs and their target genes and corresponding molecular signaling pathways together build a complex and fine network, which jointly regulate the formation of keloid [27]. MiRNA plays an important role in regulating inflammation, cancer, malignant skin diseases, wound healing, fibrotic diseases, and angiogenesis physiology, and also affects TGF- β [28]. However, if ^32^P isotope radiotherapy could regulate keloid through miRNA needs to be further explored. The abnormal expression of many genes such as epithelial mesenchymal transformation (EMT) also participates in the occurrence and development of keloid [29]. If ^32^P isotope radiotherapy could affect the EMT process remains unclear.

## CONCLUSIONS

Both ^32^P isotope injection and skin path significantly reduced the size of keloid, and the TGF-β/Smad signaling pathway was remarkably inhibited by ^32^P isotope treatment. The regulation of dermal fibroblast by ^32^P isotope was reversed by the agonist of TGF-β/Smad signaling pathway, SRI-011381. ^32^P isotope might inhibit keloid through suppressing TGF-β/Smad signaling pathway.

## MATERIALS AND METHODS

### Keloid animal model establishment

Eighteen healthy male BALB/c nude mice (Charles River, Beijing, China), aged 4–6 weeks, were selected for the experiment. Anesthesia was administered, and an 8-mm incision was made in the scapular area of each nude mouse. Keloid tissues, obtained during surgery, were cut into blocks approximately 4 mm^3^ in size and implanted in the subcutaneous space of the nude mice. The skin was then sutured to secure the implants. After 21 days, the mice were randomly divided into three groups: control, ^32^P isotope injection, and ^32^P isotope skin patch. The mice in the group ^32^P isotope skin patch were treated using the customized ^32^P isotope therapy patch from China Atomic Energy Research Institute, with an effective treatment area of 1.0 cm × 1.0 cm, with a radiation dose of 1.08 cGy/s·cm^2^. 6 layers of tin foil were used to protect the non-irradiated skin, and ^32^P isotope therapy was applied to the surface of the skin for 150 s each time. The treatment frequency is once every 2 days, with a total of 10 treatments. The gross morphology and histopathology of keloids were observed after 21 days of continuous injection. The dosage of ^32^P isotope was determined based on our pre-experiment and previous report [9].

### Cell culture

Primary dermal fibroblast cells (#PCS-201-012, ATCC, Manassas, VA, USA) were used in this research. The cells were cultured in DMEM medium (Gibco, #12491015, Langley, OK, USA) supplemented with 5% FBS, 40 μg/ml streptomycin, and 40 IU penicillin at 5% CO_2_ and 37° C. The concentration for SRI-011381 in the cell incubation was 10 μM.

### Hematoxylin-eosin (HE) staining

After sacrificing animals, the keloid was isolated, and the tissues were embedded in paraffin after dehydration. Sections of 5 μm were cut and dewaxed. The tissues were then stained with hematoxylin solution (#G1140, Solarbio, Manassas, VA, USA) for 5 minutes, washed with water until no staining solution flowed out, and incubated with PBS for 5 minutes to return to blue. Subsequently, the sections were stained with eosin (#MB9898-3, Meilunbio, Dalian, China) for 15 seconds. After decolorization with 95% ethanol for 10 seconds, the tissues were washed with water. Transparency was achieved by immersing the tissues in xylene for 10 minutes, followed by sealing with neutral gum (#MB9899, Meilunbio).

### Masson staining

Masson trichrome staining was performed as follows: The tissues were stained with hematoxylin for 10 minutes, differentiated with ethanol, incubated with Masson blue solution, and then subjected to Ponceau acid fuchsin staining for 10 minutes. After washing with phosphomolybdic acid solution for 2 minutes, aniline blue staining was conducted for 2 minutes. The tissues were then dehydrated with 95% ethanol three times for 50 seconds each, followed by incubation with xylene three times for 1 minute each. Finally, the tissues were sealed with neutral gum, and an inverted optical microscope was used to observe the stained tissues. The calculation of Masson staining was performed using Image J software.

### Reverse transcription-polymerase chain reaction (RT-PCR)

RNA was extracted from tissues using Trizol (#R0016, Beyotime, Shanghai, China), and its purity was measured with the Nanodrop 2000 spectrophotometer (Thermo Fisher Scientific, Waltham, MA, USA). The Takara PrimeScript RT reagent kit with gDNA eraser kit (#RR047A) was used for reverse transcription. RT-PCR was performed with Bio-Rad (CXF96) (Hercules, CA, USA). The relative expression level of the gene was determined using the 2^-ΔΔCT^ method. The primer sequences are provided in the following: HIF-1α (Forward 5′- CAGAAGATACAAGTAGCCTC-3′, Reverse 5′-CTGCTGGAATACTGTAACTG-3′), TGF-β (Forward 5′-GCAACAATTCCTGGCGATACCTC-3′, Reverse 5′-AGTTCTTCTCCGTGGAGCTGAAG-3′), Smad2/3 TGF-β (Forward 5′-ATGAGCACCCAGATCCAGTG-3′, Reverse 5′-CTGTGGGCTTCCTTGTTGTC-3′).

### Western blotting

Proteins were lysed using RIPA lysis buffer (Sigma-Aldrich, R0278, St. Louis, MO, USA) containing a protein phosphatase inhibitor. The protein concentration was determined using the BCA method (#A045-4-1, Nanjing Jiancheng Bioengineering Institute, Nanjing, China), and equal amounts of protein from each group were subjected to 10% SDS-PAGE. The proteins were then transferred to a PVDF membrane (Millipore, GVWP02500, Burlington, MA, USA). The membrane was blocked with 5% non-fat milk in TBST (Beyotime, #P0222) for 2 hours. Next, the membrane was incubated with primary antibodies overnight at 4° C and with secondary antibodies for 2 hours at room temperature. Finally, an enhanced chemiluminescence detection kit (Thermo Fisher Scientific) was used to detect the target genes, and ImageJ software was utilized to analyze the bands. The antibodies used in the study are listed below: HIF-1α (ab92498, Abcam, Cambridge, UK), TGF-β (ab215715, Abcam), Smad2/3 (ab232326, Abcam), p-Smad2/3 (phospho T8, ab272332, Abcam), Col1α1 (ab270946, Abcam), Col3α1 (M50852, Zenbio, Durham, NC, USA), GAPDH (ab9485, Abcam).

### Flow cytometry

Cells in the logarithmic growth phase were washed three times with PBS (#10010023, Gibco), trypsinized (#108444, Sigma-Aldrich), suspended in serum-free medium, and adjusted to a cell concentration of 1×10^7^/ml. A 100 μL cell suspension was added to a 1.5 mL microcentrifuge tube, followed by addition of 100 μL Annexin V (Beyotime, #C1062L) and PI. The cells were then incubated at room temperature in the dark for 20 minutes and analyzed using the Guava® Muse® Cell Analyzer.

### Statistical analysis

SPSS 24.0 statistical software was used for analysis, with econometric data expressed as mean ± standard deviation (x ± s). t-tests and analysis of variance were used for inter group differences analysis; count data comparison was done using χ 2 Inspection. Correlation analysis was done using Spearman correlation coefficient test. The results showed a statistically significant difference with P<0.05.

### Availability of data and material

The data and material used to support the findings of this study are included within the manuscript and supplementary files.

## References

[1] Chiu LL, Sun CH, Yeh AT, Torkian B, Karamzadeh A, Tromberg B, Wong BJ. Photodynamic therapy on keloid fibroblasts in tissue-engineered keratinocyte-fibroblast co-culture. Lasers Surg Med. 2005; 37:231–44. 10.1002/lsm.20213 16127672

[2] Leszczynski R, da Silva CA, Pinto AC, Kuczynski U, da Silva EM. Laser therapy for treating hypertrophic and keloid scars. Cochrane Database Syst Rev. 2022; 9:CD011642. 10.1002/14651858.CD011642.pub2 36161591 PMC9511989

[3] Mineda K, Sato K, Nakahara T, Minami K, Yamashita Y, Ishida S, Abe Y, Hashimoto I. Cyclical Stretching Induces Excess Intracellular Ca2+ Influx in Human Keloid-Derived Fibroblasts *In Vitro*. Plast Reconstr Surg. 2023; 151:346–54. 10.1097/PRS.0000000000009843 36696319

[4] Sun P, Hu Z, Pan B, Lu X. Targeting of keloid with TRAIL and TRAIL-R2/DR5. J Dermatolog Treat. 2021; 32:957–64. 10.1080/09546634.2020.1714541 31916474

[5] Huang F, Zhang E, Lei Y, Yan Q, Xue C. Tripterine inhibits proliferation and promotes apoptosis of keloid fibroblasts by targeting ROS/JNK signaling. J Burn Care Res. 2023. [Epub ahead of print]. 10.1093/jbcr/irad106 37436955

[6] Memariani H, Memariani M, Moravvej H, Shahidi-Dadras M. Emerging and Novel Therapies for Keloids: A compendious review. Sultan Qaboos Univ Med J. 2021; 21:e22–33. 10.18295/squmj.2021.21.01.004 33777420 PMC7968901

[7] Cockburn KC, Moore CS, Wright GA. Estimation of skin dose rate from a catheter bag filled with P-32 solution. Nucl Med Commun. 2015; 36:651–2. 10.1097/MNM.0000000000000295 25738558

[8] Zhang J, Li Y, Wen G, Deng Y, Yao H. Novel Application of 32P Brachytherapy: Treatment of Angiolymphoid Hyperplasia with Eosinophilia in the Right Auricle with 8-Year Follow-Up. Cancer Biother Radiopharm. 2018; 33:282–4. 10.1089/cbr.2018.2468 29957026 PMC6148720

[9] Vivante H, Salgueiro MJ, Ughetti R, Nicolini J, Zubillaga M. 32P-patch contact brachyradiotherapy in the management of recalcitrant keloids and hypertrophic scars. Indian J Dermatol Venereol Leprol. 2007; 73:336-9. 10.4103/0378-6323.35736 17921616

[10] Zhang T, Wang XF, Wang ZC, Lou D, Fang QQ, Hu YY, Zhao WY, Zhang LY, Wu LH, Tan WQ. Current potential therapeutic strategies targeting the TGF-β/Smad signaling pathway to attenuate keloid and hypertrophic scar formation. Biomed Pharmacother. 2020; 129:110287. 10.1016/j.biopha.2020.110287 32540643

[11] Lei R, Li J, Liu F, Li W, Zhang S, Wang Y, Chu X, Xu J. HIF-1α promotes the keloid development through the activation of TGF-β/Smad and TLR4/MyD88/NF-κB pathways. Cell Cycle. 2019; 18:3239–50. 10.1080/15384101.2019.1670508 31645185 PMC6927730

[12] Wang XM, Liu XM, Wang Y, Chen ZY. Activating transcription factor 3 (ATF3) regulates cell growth, apoptosis, invasion and collagen synthesis in keloid fibroblast through transforming growth factor beta (TGF-beta)/SMAD signaling pathway. Bioengineered. 2021; 12:117–26. 10.1080/21655979.2020.1860491 33315500 PMC8806324

[13] Wu J, Fang L, Cen Y, Qing Y, Chen J, Li Z. MiR-21 Regulates Keloid Formation by Downregulating Smad7 via the TGF-β/Smad Signaling Pathway. J Burn Care Res. 2019; 40:809–17. 10.1093/jbcr/irz089 31184708

[14] Nagar H, Kim S, Lee I, Kim S, Choi SJ, Piao S, Jeon BH, Oh SH, Kim CS. Downregulation of CR6-interacting factor 1 suppresses keloid fibroblast growth via the TGF-β/Smad signaling pathway. Sci Rep. 2021; 11:500. 10.1038/s41598-020-79785-y 33436666 PMC7804403

[15] Al-Attar A, Mess S, Thomassen JM, Kauffman CL, Davison SP. Keloid pathogenesis and treatment. Plast Reconstr Surg. 2006; 117:286–300. 10.1097/01.prs.0000195073.73580.46 16404281

[16] Trace AP, Enos CW, Mantel A, Harvey VM. Keloids and Hypertrophic Scars: A Spectrum of Clinical Challenges. Am J Clin Dermatol. 2016; 17:201–23. 10.1007/s40257-016-0175-7 26894654

[17] Kuo YR, Wu WS, Wang FS. Flashlamp pulsed-dye laser suppressed TGF-beta1 expression and proliferation in cultured keloid fibroblasts is mediated by MAPK pathway. Lasers Surg Med. 2007; 39:358–64. 10.1002/lsm.20489 17457842

[18] Pires JA, Bragato EF, Momolli M, Guerra MB, Neves LM, de Oliveira Bruscagnin MA, Ratto Tempestini Horliana AC, Porta Santos Fernandes K, Kalil Bussadori S, Agnelli Mesquita Ferrari R. Effect of the combination of photobiomodulation therapy and the intralesional administration of corticoid in the preoperative and postoperative periods of keloid surgery: A randomized, controlled, double-blind trial protocol study. PLoS One. 2022; 17:e0263453. 10.1371/journal.pone.0263453 35167583 PMC8846523

[19] Scrimali L, Lomeo G, Tamburino S, Catalani A, Perrotta R. Laser CO2 versus radiotherapy in treatment of keloid scars. J Cosmet Laser Ther. 2012; 14:94–7. 10.3109/14764172.2012.671524 22384790

[20] Dong W, Qiu B, Fan F. Adjuvant Radiotherapy for Keloids. Aesthetic Plast Surg. 2022; 46:489–99. 10.1007/s00266-021-02442-w 34415398

[21] Mankowski P, Kanevsky J, Tomlinson J, Dyachenko A, Luc M. Optimizing Radiotherapy for Keloids: A Meta-Analysis Systematic Review Comparing Recurrence Rates Between Different Radiation Modalities. Ann Plast Surg. 2017; 78:403–11. 10.1097/SAP.0000000000000989 28177974

[22] Sakamoto T, Oya N, Shibuya K, Nagata Y, Hiraoka M. Dose-response relationship and dose optimization in radiotherapy of postoperative keloids. Radiother Oncol. 2009; 91:271–6. 10.1016/j.radonc.2008.12.018 19201502

[23] Wang W, Qu M, Xu L, Wu X, Gao Z, Gu T, Zhang W, Ding X, Liu W, Chen YL. Sorafenib exerts an anti-keloid activity by antagonizing TGF-β/Smad and MAPK/ERK signaling pathways. J Mol Med (Berl). 2016; 94:1181–94. 10.1007/s00109-016-1430-3 27339758 PMC5052317

[24] Hu ZC, Shi F, Liu P, Zhang J, Guo D, Cao XL, Chen CF, Qu SQ, Zhu JY, Tang B. TIEG1 Represses Smad7-Mediated Activation of TGF-β1/Smad Signaling in Keloid Pathogenesis. J Invest Dermatol. 2017; 137:1051–9. 10.1016/j.jid.2016.12.019 28108300

[25] Shi CK, Zhao YP, Ge P, Huang GB. Therapeutic effect of interleukin-10 in keloid fibroblasts by suppression of TGF-β/Smad pathway. Eur Rev Med Pharmacol Sci. 2019; 23:9085–92. 10.26355/eurrev_201910_19311 31696499

[26] Li Q, Fang L, Chen J, Zhou S, Zhou K, Cheng F, Cen Y, Qing Y, Wu J. Exosomal MicroRNA-21 Promotes Keloid Fibroblast Proliferation and Collagen Production by Inhibiting Smad7. J Burn Care Res. 2021; 42:1266–74. 10.1093/jbcr/irab116 34146092

[27] Jones LR, Levin AM, Dai X, Datta I, Li J, Yin C, Mi QS. MicroRNA Profile Differentiates Head and Neck Keloid and Adjacent Normal Skin Tissue. Facial Plast Surg Aesthet Med. 2021. [Epub ahead of print]. 10.1089/fpsam.2020.0414 33710934 PMC11271068

[28] Yan L, Wang LZ, Xiao R, Cao R, Pan B, Lv XY, Jiao H, Zhuang Q, Sun XJ, Liu YB. Inhibition of microRNA-21-5p reduces keloid fibroblast autophagy and migration by targeting PTEN after electron beam irradiation. Lab Invest. 2020; 100:387–99. 10.1038/s41374-019-0323-9 31558773

[29] Lee YI, Shim JE, Kim J, Lee WJ, Kim JW, Nam KH, Lee JH. WNT5A drives interleukin-6-dependent epithelial-mesenchymal transition via the JAK/STAT pathway in keloid pathogenesis. Burns Trauma. 2022; 10:tkac023. 10.1093/burnst/tkac023 36225328 PMC9547497

